# Cultured Horse Articular Chondrocytes in 3D-Printed Chitosan Scaffold With Hyaluronic Acid and Platelet Lysate

**DOI:** 10.3389/fvets.2021.671776

**Published:** 2021-07-12

**Authors:** Elena De Angelis, Roberta Saleri, Paolo Martelli, Lisa Elviri, Annalisa Bianchera, Carlo Bergonzi, Marta Pirola, Roberta Romeo, Melania Andrani, Valeria Cavalli, Virna Conti, Ruggero Bettini, Benedetta Passeri, Francesca Ravanetti, Paolo Borghetti

**Affiliations:** ^1^Department of Veterinary Science, University of Parma, Parma, Italy; ^2^Food and Drug Department, University of Parma, Parma, Italy

**Keywords:** chitosan, platelet lysate, chondrocytes, cartilage, biomaterials, tissue engineering, hyaluronic acid

## Abstract

Three-dimensional (3D) printing has gained popularity in tissue engineering and in the field of cartilage regeneration. This is due to its potential to generate scaffolds with spatial variation of cell distribution or mechanical properties, built with a variety of materials that can mimic complex tissue architecture. In the present study, horse articular chondrocytes were cultured for 2 and 4 weeks in 3D-printed chitosan (CH)-based scaffolds prepared with or without hyaluronic acid and in the presence of fetal bovine serum (FBS) or platelet lysate (PL). These 3D culture systems were analyzed in terms of their capability to maintain chondrocyte differentiation *in vitro*. This was achieved by evaluating cell morphology, immunohistochemistry (IHC), gene expression of relevant cartilage markers (*collagen type II, aggrecan, and Sox9*), and specific markers of dedifferentiated phenotype (*collagen type I, Runx2*). The morphological, histochemical, immunohistochemical, and molecular results demonstrated that the 3D CH scaffold is sufficiently porous to be colonized by primary chondrocytes. Thereby, it provides an optimal environment for the colonization and synthetic activity of chondrocytes during a long culture period where a higher rate of dedifferentiation can be generally observed. Enrichment with hyaluronic acid provides an optimal microenvironment for a more stable maintenance of the chondrocyte phenotype. The use of 3D CH scaffolds causes a further increase in the gene expression of most relevant ECM components when PL is added as a substitute for FBS in the medium. This indicates that the latter system enables a better maintenance of the chondrocyte phenotype, thereby highlighting a fair balance between proliferation and differentiation.

## Introduction

Osteoarthritis (OA) is one of the major challenges in joint pathology in both humans and animals. Once damaged, the cartilage has limited capability for repair due to its avascular nature and to the low metabolic index of chondrocytes. Cellular and molecular mechanisms that cause altered repair involve the loss of chondrocyte differentiation and cartilage homeostasis (1). In addition, fibrocartilaginous tissue is formed, with reduced mechanical capacity compared with native cartilage.

Tissue engineering is a potential field of regenerative medicine. It is based on the interaction of three main elements (a support biomaterial, growth factors, and cells) for the development of a biological substitute that can be used for replacement, restoration, or regeneration of damaged tissues and organs (2).

The use of live cells and biocompatible polymers represents an important technique for the repair of cartilage defects. This is primary considering that the seeding of chondrocytes onto these three-dimensional (3D) scaffolds is the main condition for maintaining chondrocyte differentiation and cartilage ECM synthesis (3, 4). The selected biomaterial sustaining chondrocyte cell growth must exhibit several characteristics in relation to the chemical and physical properties of the native cartilage (5, 6).

Accordingly, a porous polymeric scaffold provides a temporary substrate that guides and sustains cell proliferation, differentiation, and tissue growth. Moreover, a mechanically active scaffold plays an important role in the maintenance of structural integrity and delivery of signals that stimulate cell adhesion and synthesis. Studies have been carried out on horses to assess the suitability of chondrocyte transplantation for the repair of articular cartilage defects. A few of these verified that chondrocyte implantation into a biocompatible matrix can result in a promising therapeutic option for the treatment of cartilage defects (7–11).

Among the suitable biomaterials for the maintenance of differentiated chondrocytes and for the delivery of cells into a tissue, chitosan (CH) and hyaluronic acid (HA), are two polysaccharides that are widely utilized as scaffolds in articular cartilage regeneration (12).

CH has a structure like that of glycosaminoglycans (GAGs) of the extracellular matrix (ECM) of native cartilage (13, 14). Furthermore, it has been recommended as a good biomaterial for the growth and maintenance of chondrocytes *in vitro* (8, 15–18).

Hyaluronate is a GAG, natural component of cartilage ECM and of the synovial fluid of joints. In cartilage, HA links aggrecan monomers, to form large aggregating proteoglycans (LAPs); LAPs retain water and create an “internal swelling pressure” that counteracts compression forces in articular cartilage (19). HA also functions as an environmental cue to regulate cell behavior during embryonic development, healing processes, and inflammation (20).

In addition, *in vitro* and *in vivo* studies have shown that platelet-rich plasma (PRP) has positive effects on articular cartilage (21–26). *In vivo*, it has been reported that intra-articular injection of autologous PL aids in temporary management of OA of the distal interphalangeal joint in athletic horses (27) and significantly improves clinical scores of OA in human patients (28). *In vitro* studies have reported that PRP induces proliferation and synthesis of ECM components in chondrocytes (29–31), and chondrogenesis (32).

This effect is due to several growth factors that exert trophic effects on cartilage (30, 33–37). Platelet lysate (PL) is obtained from PRP by the release of growth factors from platelets through repeated freezing and thawing cycles. It has recently been studied as an alternative to fetal bovine serum (FBS) in culture (38–40).

The manufacturing of scaffolds and/or constructs through 3D printing has gained popularity in tissue engineering owing to its capability to develop complex formulations for cartilage regeneration (41–44).

Recently, the behavior of 3D-printed CH-based scaffolds was explored as a function of the post-printing gelation process, in terms of the capability to retain the 3D structure, water content, mechanical resistance, and surface/internal porosity, as well as the biocompatibility with fibroblasts as a skin-associated human cell line (45).

We propose that 3D-printed CH-based scaffolds represent a suitable environment for chondrocyte culture *in vitro* and that its enrichment with HA could be helpful for the maintenance of chondrocyte phenotype. Moreover, the supplementation with PL could improve the phenotype stability of chondrocytes better then FBS, as observed in chondrocytes in adherent conditions (39, 46). In the present work, 3D-printed CH scaffold was prepared with HA and studied in terms of its capability to maintain chondrocyte differentiation *in vitro* in the presence of FBS or PL.

## Materials and Methods

### Preparation of 3D Chitosan Scaffolds

Chitosan powder (ChitoClear™ TM4030, DD of 75%; MW 50 kDa, Primex Ehf, Iceland) was dispersed at the concentration of 6% w/v into a 0.02% w/v aqueous solution of sodium hyaluronate (HA) (Halien, High MW, ACME s.r.l., Cavriago, Italy, batch Ha16003), magnetically stirred until homogenization (5 s) and made up to volume dropwise with glacial acetic acid (Sigma-Aldrich) until a final concentration of 2% v/v. Complete dissolution of chitosan was achieved after overnight magnetic stirring at 24°C. D-(+)-raffinose pentahydrate (Sigma-Aldrich, USA) was added at a concentration of 290 mM as viscosity agent (47) and dissolved by magnetic stirring. The same procedure was adopted to prepare the chitosan-based solution free of hyaluronic acid: in this case, dissolution of chitosan took place in a 2% v/v acetic acid solution in UltraPure water (Purelab Flex 1 system, ELGA Veolia) to which D-(+)-raffinose was added at 290 mM. Polymeric blends were stored at 4°C until use.

The CAD model for 3D printing of scaffolds was designed by means of Solidworks™ software (Dassault Systems, USA) by drawing a grid composed of overlapping parallel filaments set at a nominal distance of 200 μm. Total sizes of the object, formed by five layers, were also set, 1.5 × 1.5 × 0.1 cm (width/length/thickness). The file produced was then converted through the software Slic3r™ (RepRap) from the generated. stl format (STereo Lithography interface format) to the machine code (gcode format) readable by the in-house-built 3D printer. Briefly, the platform physically supporting the 3D construction consisted of a 0.6-mm-thick stainless-steel plate coated with a 0.6-mm chitosan or chitosan–HA film casted prior to printing, which was fixed on the Peltier cells of the 3D machinery and instantly frozen at temperature of −18°C: this film constituted the base for the scaffold. Polymeric blends were loaded into 5-ml syringes equipped with a blunt 26 G needle (internal ø: 0.260 mm), which were fixed on the machinery's robotic arms and connected to the pressure-assisted mechanical piston (extruder). Solutions were deposited by the extruding apparatus moving along *x*-, *y*-, and *z*-axis on the top of the base film, and instantly frozen at a constant temperature of −18°C exploiting freeze deposition layer by layer to give shape to the polymeric blend to obtain the 3D object designed, according to a method recently described (48). At the end of each manufacturing process, frozen scaffolds underwent ionotropic gelation by exposure to saturated ammonia vapors for 1 h at ambient temperature, following the technique reported in a previous work dealing with the settlement of suitable gelation processes for 3D chitosan hydrogels (45). Scaffolds were then washed by immersion in 80 ml of UltraPure water until neutral pH (four times for 10 min) to eliminate ammonia residues and raffinose (47). Finally, scaffolds were punched round to fit into 24-well plates, sterilized by immersion in 70% w/v ethanol, transferred to sterile phosphate buffered saline (PBS) and preserved at 4°C until use.

The mechanical resistance of scaffolds obtained by different gelation media was compared on 20-layer scaffolds of 5 × 1.5 cm. Hydrogels at the maximum swollen state were tested. Thickness was determined as a mean of six measurements of the scaffold performed with a digital micrometer (Mitutoyo, Japan). Each scaffold was fixed on a tensile tester (AG M1 Acquati, Italy) loaded with a 5-daN cell. Force and time signals were digitalized by means of a PowerLab 400 board and recorded with Scope 3.5 software. Elongation at break (% strain) and Young's modulus were determined from the relevant stress–strain curves, taking into consideration the linear portion and normalized by thickness (45).

### Preparation of PL

The donor horses were referred to the Veterinary Teaching Hospital of the University of Parma and were examined by a veterinarian to assess their clinical history and to exclude systemic and hematological diseases. Blood was collected and placed into tubes supplemented with sodium citrate (0.38% final concentration). The citrated blood was centrifuged in a standard laboratory centrifuge (swinging rotor) for 20 min at 180 × *g*. Afterwards, the portion of plasma enriched with platelets was transferred to another tube and platelets were pelleted in a second centrifugation step for 15 min at 900 × *g*. The pellet was resuspended so as to obtain a concentration of 1 × 10^9^ platelets per 1 ml. The PRP was subjected to a double freezing cycle at −80°C and thawing at −37°C. To remove platelet remnants derived from freezing–thawing cycles, a further centrifugation was performed (Microfuge 22R Centrifuge) for 20 min at 11,000 × *g*. PL supernatant was collected and stored at −80°C until use (39).

### Cell Cultures

Horses ranging from 5 to 8 years old and farmed for human consumption were slaughtered and the metacarpophalangeal and metatarsophalangeal joints were immediately delivered to our laboratory for collection of cartilage. Before cartilage harvesting, joints were carefully examined and those with macroscopic lesions related to overt OA or with evidence of synovitis were excluded from the study. The articular cartilage was obtained from three joints/experiment; chondrocytes were isolated separately from each cartilage and then checked for viability and the samples with viability <95% were excluded. Then, three samples were pooled for each experiment and three experiments were done, with three replications/experiment.

Chondrocytes were isolated from tissue following a protocol previously described (39). Briefly, cartilage was finely diced under sterile conditions and then washed several times in PBS. After pre-incubation in 0.1% pronase (Sigma) solution for 1 h at 37°C, the tissue was treated with 0.2% collagenase type IA (Sigma) in D-MEM (4.5 g/L glucose; 25 mM Hepes) for 2 h at 37°C. The digested material was filtered through 100-μm and 20-μm nylon filters and the cellular suspension was centrifuged at 1,800 rpm for 10 min. The supernatant was discarded, and the pellet was washed several times with D-MEM containing 10% fetal calf serum (FBS), 100 U/ml penicillin and 0.1 mg/ml streptomycin. The number of chondrocytes was determined using a hemocytometer and the cell viability (never <95%) was assessed by trypan blue (0.1%) exclusion. The chondrocytes were seeded into 3D chitosan scaffolds (CH) with or without hyaluronic acid (CH+HA) at a density of 8 × 10^5^ cells/scaffold.

The culturing medium contained 10% FBS or 5% PL or nothing (–FBS/–PL). All cultures were incubated at 37°C in a humidified atmosphere of 5% CO_2_ in air for the time specified for each single experiment and the medium was exchanged biweekly.

### Histology

The adhesion, colonization, and morphological characteristics of the cells in 3D systems was histologically evaluated after 2 weeks and 4 weeks of culture.

At the end of experimental time, scaffolds were fixed with 4% paraformaldehyde (PFA) in PBS for 40 min at room temperature. After fixation, the scaffolds were washed to eliminate any residues of PFA and then pre-embedded in 100 μl of 2% agarose solution in distilled water to preserve the 3D culture and facilitate scaffold handling. The samples were then dehydrated in increasing alcohol concentrations, clarified in xylene, and paraffin embedded. Histological sections 5 μm thick were obtained using a rotary microtome (Slee Cut 6062, Slee Medical, Mainz, Germany). Sections were stained with hematoxylin–eosin (H&E) and Masson's trichrome (with Aniline-Blue). Microphotographs were acquired with Nikon Digital Sight System.

### Immunohistochemistry

Immunohistochemical analysis was performed at 2 and 4 weeks of culture in order to determine the positivity of ECM to collagen type II. Sections were treated with 2% Hyaluronidase (Sigma) in PBS for 30 min at 37°C. Endogenous peroxidases were quenched with 3% H_2_O_2_ for 12 min at room temperature and then aspecific antigens were blocked with 1% bovine serum albumin (BSA) (Sigma) in PBS for 1 h at room temperature. The slides were then incubated overnight at a 4°C with primary monoclonal antibody anti-collagen II (1:100) (orb 156420; Byorbit). Then, secondary biotinylated anti-rabbit antibody (BA-100, Vector Laboratories) was applied for 1 h at room temperature. Slides were incubated with an ABC Kit (PK7800, Vector laboratories), and then with 3,3′-diaminobenzidine (DAB) for 3 min, counterstained with Mayer's hematoxylin and then dehydrated in ascending scale of alcohols and permanently mounted.

Slides were then digitalized and collagen type II was quantified using NIS-elements AR (Nikon, Japan) on three (per case) digital images at 10 × magnification. The total number of cells was obtained identifying the nuclei through a nuclear threshold-based mask; this mask was converted into regions of interest (ROIs) (each nucleus represents a single counting ROI). In order to include cytoplasm and pericellular space, the mask was expanded for three layers through a mathematical morphology-thickening function (NIS–element AR, Nikon, Japan). The number of positive cells [(N of collagen Type II positive cells/total number of cells) * 100] was obtained overlaying the ROIs with a threshold based on collagen type II signal and considering only ROIs with positive signaling.

### Scanning Electron Microscopy

The scanning electron microscopy (SEM) of cells grown on chitosan scaffolds was performed after 2 weeks and 4 weeks of culture (49, 50). Scaffolds seeded with cells were fixed at each selected time point with 2.5% glutaraldehyde in 0.1 M sodium cacodylate buffer (pH 7.3) for 2 h at 4°C. They were then dehydrated through a series of increasing grades of ethanol (from 50% v until absolute) and then critical point dried with liquid carbon dioxide (CPD 030 Baltec, Wallruf, Germany). Specimens were then sputter-coated (Balzers device) with gold-palladium (Plano, Germany) using a SCD 040 coating device (Balzer Union, Wallruf, Germany). The samples were observed using a Philips 501 SEM scanning electron microscope at an accelerating voltage of 5 kV. The chitosan scaffolds without cells were used as control samples (blank).

### Gene Expression of Differentiation Markers

#### Total RNA Extraction

Total RNA of each sample was extracted using combined protocol with TRIreagent (Thermo Fisher Scientific) and PureLink® RNA Micro Kit (Thermo Fisher Scientific), according to the manufacturer's instructions. Scaffolds containing cells were dissolved into TRIreagent and then the RNA was extracted according to the datasheet of PureLink® RNA Micro Kit. All RNA samples were DNase-treated during protocol execution. Purity and concentration were assessed by UV spectrophotometry at 260/280 and 260 nm, respectively (BioSpectrometer, Eppendorf, Hamburg, Germany). RNA integrity and quality were assessed by using an Agilent Bioanalyzer 2100 and RNA 6000 Labchip kit (Agilent Technologies, Santa Clara, CA, USA).

#### Reverse Transcription (RT)

Total RNA (1 μg/20 μl) was reverse-transcribed using a High-capacity cDNA Reverse Transcription kit (Applied Biosystems–Life Technologies, Carlsbad, CA, USA) according to the manufacturer's instructions, under the following thermal conditions performed by using a StepOne thermocycler (Applied Biosystems, StepOne): 10 min at 25°C, 120 min at 37°C, followed by 5 min at 85°C. All cDNA samples were stored at −20°C until PCR was performed.

#### Quantification of mRNA by Real-Time PCR (qPCR)

cDNA concentration was assessed by UV spectrophotometry (BioSpectrometer, Eppendorf, Hamburg, Germany) and 5 ng of each sample was used as a template for real-time quantitative PCR (qPCR) performed by using a StepOne thermocycler (Applied Biosystems, StepOne software v. 2.1). The cDNA (5 ng/20 μl) was amplified in triplicate with Power Up SYBR Green Master Mix (Applied Biosystems-Life Technologies, Carlsbad, CA, USA) along with specific sets of primers at 300 or 500 nM. The primers were designed based on published gene sequences or by using Primer Express® software package (Applied Biosystems) to create oligonucleotides with similar melting temperatures and minimal self-complementary, and purchased from Eurofins Genomics (Ebersberg, Germany). Details of each primer set for detection of gene expression are reported in Table 1 (39).

**Table 1:** Primer sequences used for real-time polymerase chain reaction.

**Gene**	**Primer sequences**	**Primer concentration (nM)**
*GAPDH*	FWD: CAAGGCTGTGGGCAAGGT	300
	REV: GGAAGGCCATGCCAGTGA	300
*COL1A1*	FWD: AGAAGAAGACATCCCAGCAGTCA	500
	REV: CAGGGCTCGGGTTTCCATA	500
*COL2A1*	FWD: CTGGTGATGATGGTGAAG	300
	REV: GTAACCTCTGTGACCTTTG	300
*ACAN*	FWD: GACCACTTTACTCTTGGCGTTTG	500
	REV: GTCAGGGTCTGAAACGTCTACTGA	500
*VCAN*	FWD: GCAACCCATGCACTACATAAAGTC	500
	REV: TCCAGAGAGGGAGCCCTTAAC	500
*SOX9*	FWD: CAGGTGCTCAAGGGCTACGA	300
	REV: GACGTGAGGCTTGTTCTTGCT	300
*RUNX2*	FWD: CCCGTGGCCTTCAAAGTG	300
	REV: TGACAGTAACCACAGTCCCATCTG	300

*GAPDH, glyceraldehyde-3-phosphate dehydrogenase; COL1A1, Collagen type I; COL2A1, Collagen type II; ACAN, Aggrecan; VCAN, Versican; SOX9, Transcription factor SOX-9; RUNX2, Runt-related transcription factor 2*.

The reference gene *GAPDH (glyceraldehyde-3-phosphate dehydrogenase)* was selected as an endogenous control according to minimal intra-/inter-assay variation. Samples were kept at 95°C for 20 s (hold step) to allow DNA-polymerase activation and then subjected to 40 cycles consisting of a denaturation step at 95°C for 3 s followed by an annealing/extension step at 60°C for 30 s. Fluorescence due to SYBR Green I incorporation was acquired at the end of the extension step. A no-RT control and a no-template control (NTC) were included in each experiment. A melting curve analysis for specific amplification control was performed (from 60 to 95°C) at the end of the amplification cycles. NTC controls were assumed as negative and reliable if the quantification cycle (Cq) was ≥35. Data were analyzed according to the 2^−ΔΔCt^ method (51, 52) in which expression levels of each gene are normalized to the *GAPDH* cDNA amount and expressed as relative quantities.

### Statistical Analysis

The experimental results were presented as the mean ± SD. In each experiment, each treatment was performed with three replicate culture wells. Statistical differences were calculated with multifactorial ANOVA using the SPSS 21.0 for Window (IBM® SPSS® Statistics, IBM, New York, USA). When significant differences were found, means were compared by Scheffe's *F* test.

## Results

### Scaffold Structure and Cell Colonization

**Figure 1:**
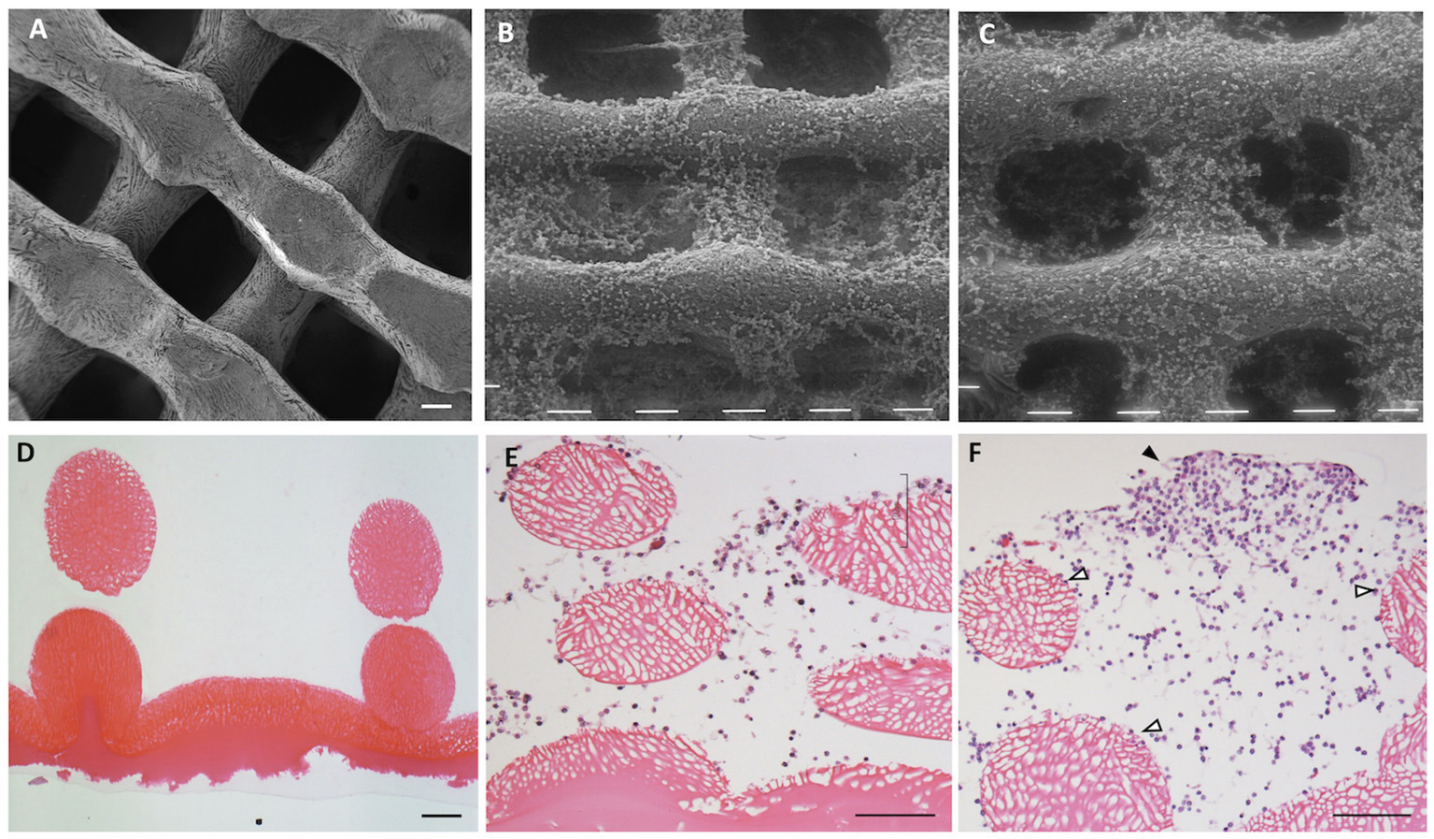
Chitosan scaffold at scanning electron microscopy (SEM) **(A–C)** and at light microscopy (hematoxylin–eosin staining; **D–F**). The structure of chitosan scaffold without cells **(A,D)**. Chitosan scaffold **(B,E)** and chitosan scaffold functionalized with hyaluronic acid (HA) **(C,F)** with chondrocytes after 2 weeks of culture. Black tips: cell cluster; white tips: single cells. Scale bar = 100 μm.

The structure of the 3D CH scaffold consisted of an upper part of a regular network of CH fibers forming pores of approximately 200 μm (Figure 1A) and a basal layer of CH film (Figure 1D) to hold cells and prevent their adhesion to the plastic plate.

Mechanical resistance was tested for both types of scaffold, resulting in 128 kPa ± 21 kPa (% strain at rupture 52.3 ± 5.3%) for the chitosan scaffold and 156 kPa ± 25 kPa (% strain at rupture 50.6 ± 10.9%) for the chitosan + hyaluronic acid scaffold, without any statistically significant difference. Furthermore, the scaffolds retained their structure for up to 6 months when stored in water or saline solution: no statistically significant alteration of the structure was observed. When immersed in culture media, all scaffolds retained their original dimensions for up to 21 days.

After 2 weeks of culture in both types of scaffolds (Figures 1B,C), chondrocytes colonized the scaffold reticulate. A few cells settled in the spaces within the reticulate, whereas others adhered to the surface of fibers of scaffold.

The chondrocytes penetrated inside the 3D structure and colonized the spaces between the fibers (Figure 1E). The chondrocytes used the spaces as niches for proliferation and growth by organizing in clusters, which became larger during culture (Figure 1F, black arrowhead) or remained as single cells adhering to the surface of the scaffold fibers (Figure 1F, white arrowhead).

### Scanning Electron Microscopy

**Figure 2:**
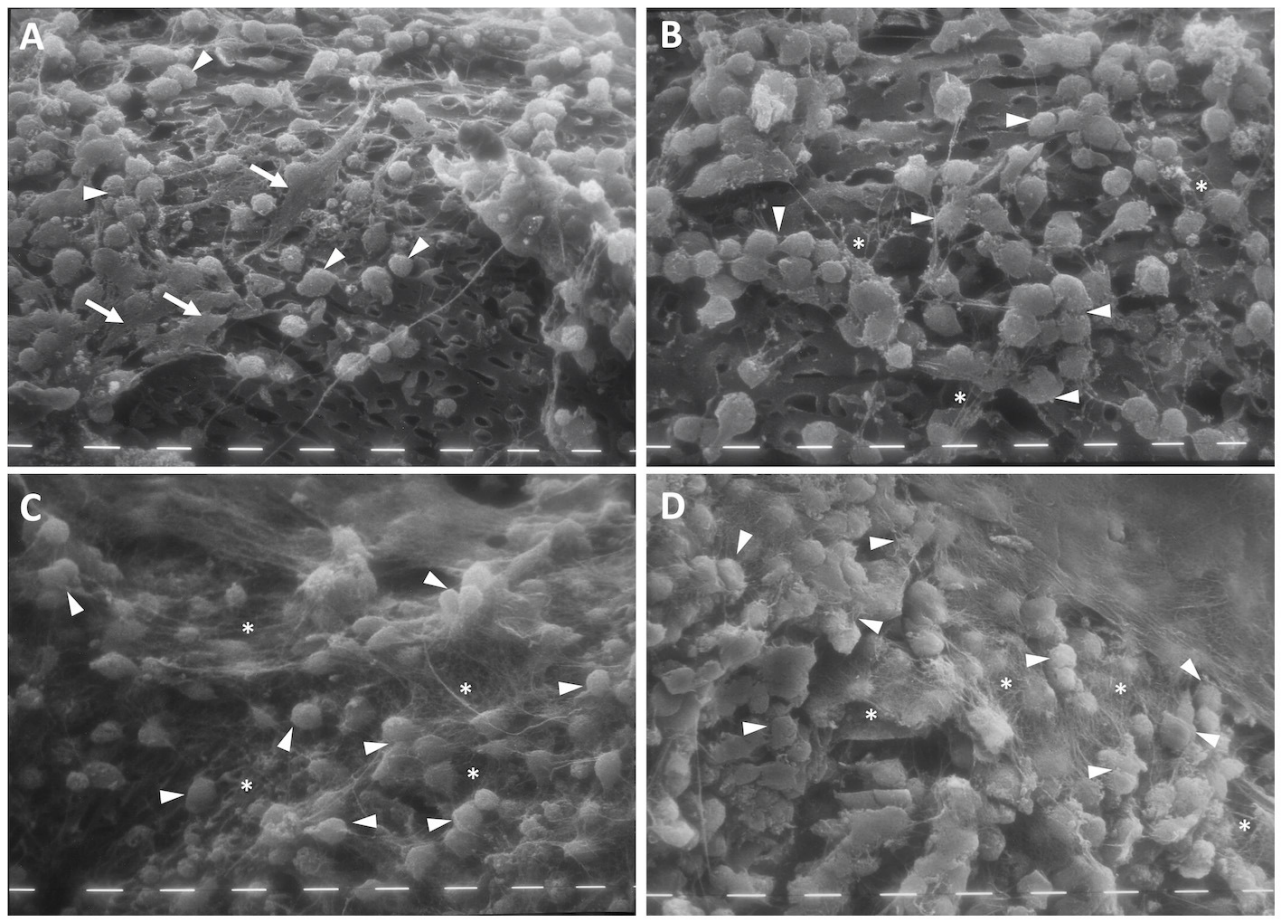
Scanning electron microscopy (SEM) of chitosan scaffold **(A,C)** and chitosan scaffold prepared with hyaluronic acid (HA) **(B,D)** with chondrocytes after 2 weeks of culture with FBS **(A,B)** and PL **(C,D)**. White arrows: fibroblast-like cells; white tips: round cells; white stars: filaments of matrix. Scale bar = 10 μm.

After 2 weeks, the chondrocytes cultured with FBS colonized the pores of the scaffold's reticulate texture. However, these appeared with an evident double cell morphology, both roundish cells and fibroblast-like cells (Figure 2A, white arrowheads and white arrows). When the scaffold was prepared with HA, roundish chondrocytes became predominant and a little production of filamentous matrix became evident (Figure 2B, white stars). In the presence of PL, the chondrocytes had a roundish morphology, and the presence of filaments compatible with matrix production was apparent in both types of scaffolds (Figures 2C,D, white stars).

**Figure 3:**
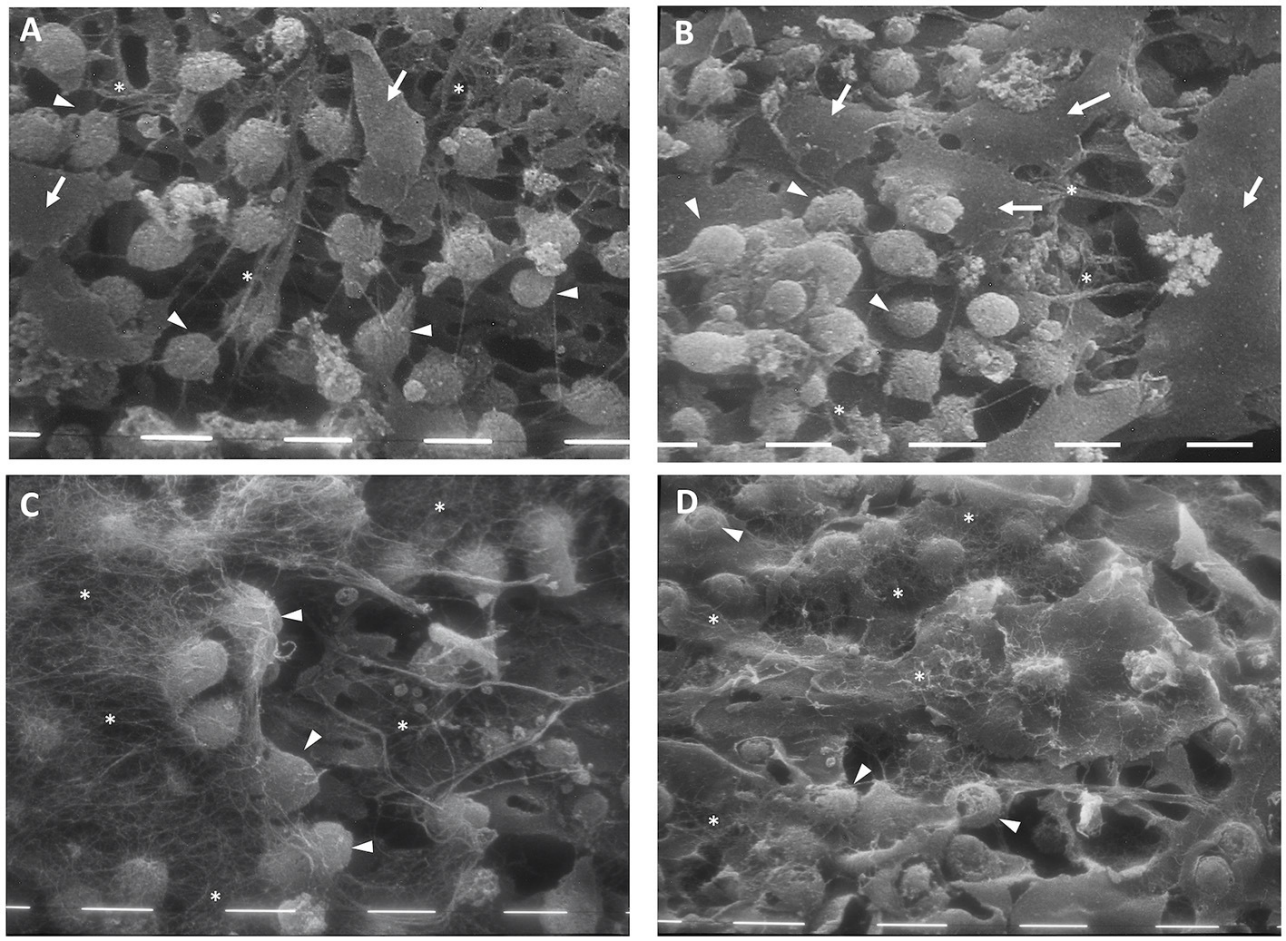
Scanning electron microscopy (SEM) of chitosan scaffold **(A,C)** and chitosan scaffold prepared with hyaluronic acid (HA) **(B,D)** with chondrocytes after 4 weeks of culture with FBS **(A,B)** and PL **(C,D)**. White arrows: fibroblast-like cells; white tips: round cells; white stars: filaments of matrix. Scale bar = 10 μm.

After 4 weeks of culture with FBS, the chondrocytes colonized the pores of the scaffold's reticulate texture. Moreover, these appeared to be more numerous in CH scaffolds both with and without HA enrichment, compared with that for PL treatment. In the FBS condition, the chondrocytes maintained the double morphological features, and a few fibroblast-like cells became evident in the scaffold prepared with HA (Figure 3B, white arrows). Furthermore, the cells began to produce a few matrix filaments (Figures 3A,B, white stars).

After 4 weeks of PL supplementation, the cell population increased and the chondrocytes that covered the scaffold texture were more numerous, compared with the scenario after 2 weeks. In this culture condition, the chondrocytes showed a round morphology and a significant amount of filamentous matrix production (Figures 3C,D, white stars) in both types of scaffolds.

### Histology

The production of ECM by the chondrocytes was evaluated using Masson's trichrome stain. It highlighted the collagen-based ECM in blue, whereas the scaffold structure and cells were stained in dark red.

**Figure 4:**
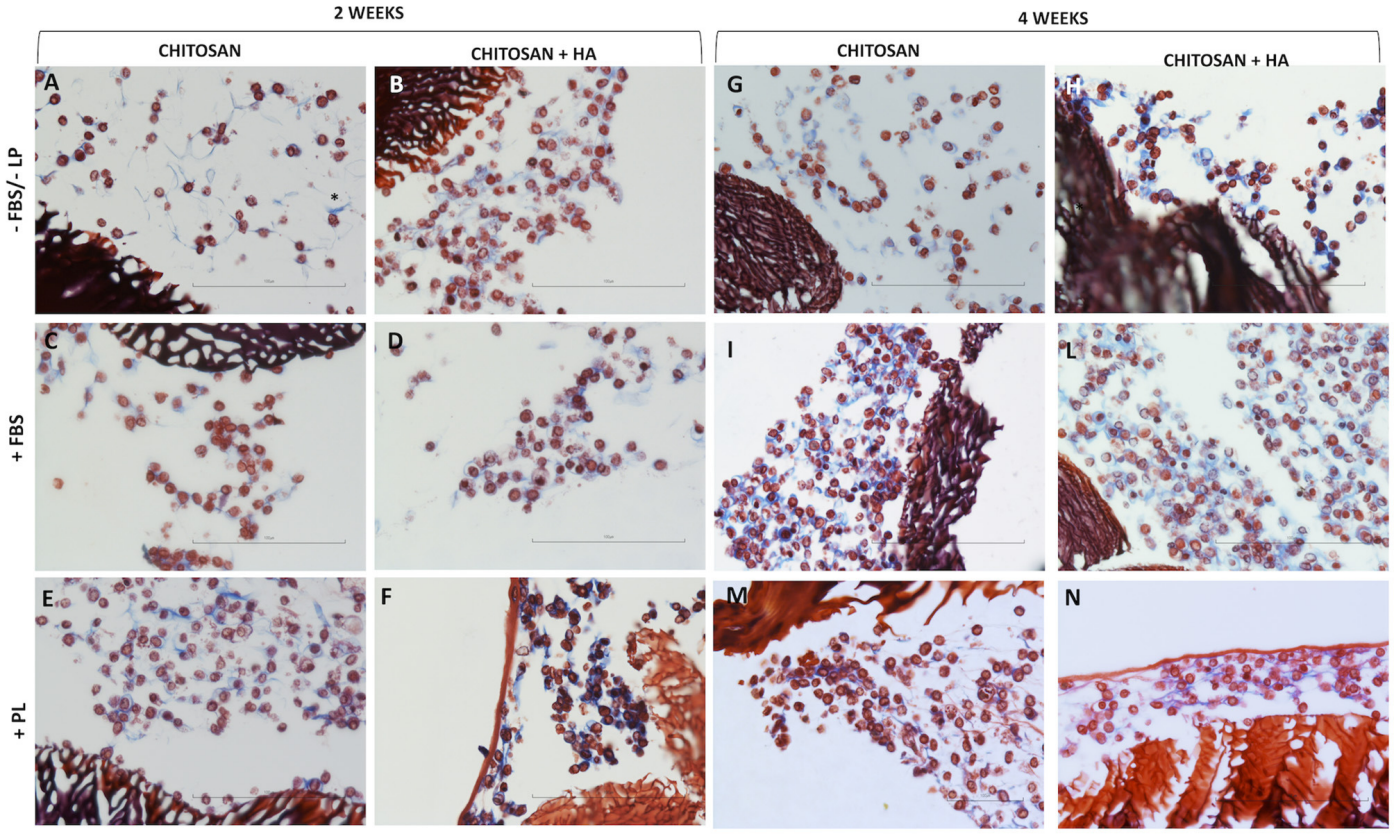
Light microscopy of chitosan scaffolds **(A,C,E)** and chitosan scaffolds prepared with hyaluronic acid (HA) **(B,D,F,H,L,N)** with chondrocytes cultured in different conditions after 2 and 4 weeks. –FBS/–PL **(A,B,G,H)**; +FBS **(C,D,I,L)**; +PL **(E,F,M,N)**. Masson's trichrome staining, Scale bar = 100 μm.

After 2 weeks of culture, matrix production was detected under all the conditions (Figure 4). However, this was more evident in the CH scaffolds with HA than in those without HA, even in the absence of FBS/PL supplementation (Figure 4B). Furthermore, the presence of PL increased matrix production in both types of scaffolds (Figures 4E,F).

After 4 weeks of culture, the cells seeded on both types of scaffolds (Figure 4, right panel) continued to present a roundish and regular morphology comparable to that observed at 2 weeks. However, higher cell density was observed in certain areas of the scaffolds in the presence of FBS and PL (Figures 4I,L–N).

The matrix production was increased compared with that at 2 weeks under all the conditions. This was particularly so in the CH scaffolds with HA without FBS/PL supplementation (Figure 4H).

### Immunohistochemistry

**Figure 5:**
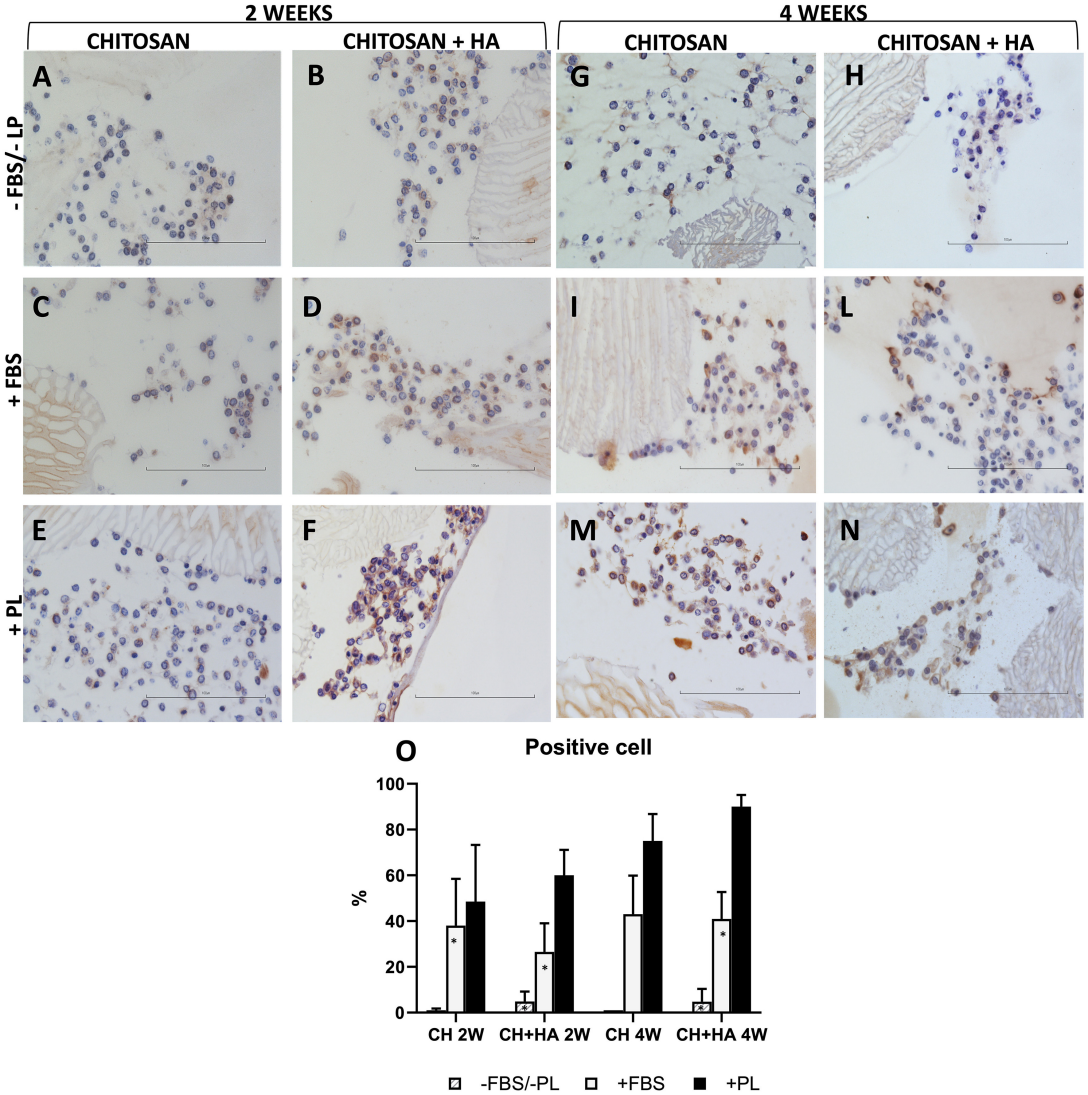
Immunohistochemical stain for collagen type II in chitosan scaffolds **(A,C,E,G,I,M)** and chitosan scaffolds prepared with hyaluronic acid (HA) **(B,D,F,H,L,N)** with chondrocytes cultured in different conditions after 2 and 4 weeks. –FBS/–PL **(A,B,G,H)**; +FBS **(C,D,I,L)**; +PL **(E,F,M,N)**. Scale bar = 100 μm. **(O)** Quantification of Collagen type II expressed as percentage of positive cell. Statistical analysis: asterisk indicate significant differences versus PL (*P < 0.05, *n* = 6).

Collagen type II was used as a marker of chondrocyte ECM production. The expression of collagen type II after 2 weeks of culture (Figure 5, left panel) was low in the scaffolds without HA under all the tested culturing conditions (Figures 5A,C,E,O). A marginal more evident collagen II positivity was observed only in the PL supplementation condition. Rather, CH scaffolds with HA showed ECM production characterized by expression of collagen type II, particularly in FBS and PL supplementation conditions (Figures 5D,F,O).

After 4 weeks (Figure 5, right panel), collagen type II positivity was also evident in the ECM of the CH scaffolds without HA (Figures 5I,M,O) and remained higher in the CH scaffold with HA (Figures 5H,I,N,O). Without FBS/PL supplementation, the collagen type II expression remained at baseline (Figures 5G,H,O).

### Gene Expression

#### Gene Expression of Col1, Col2, ACAN, and VCAN

**Figure 6:**
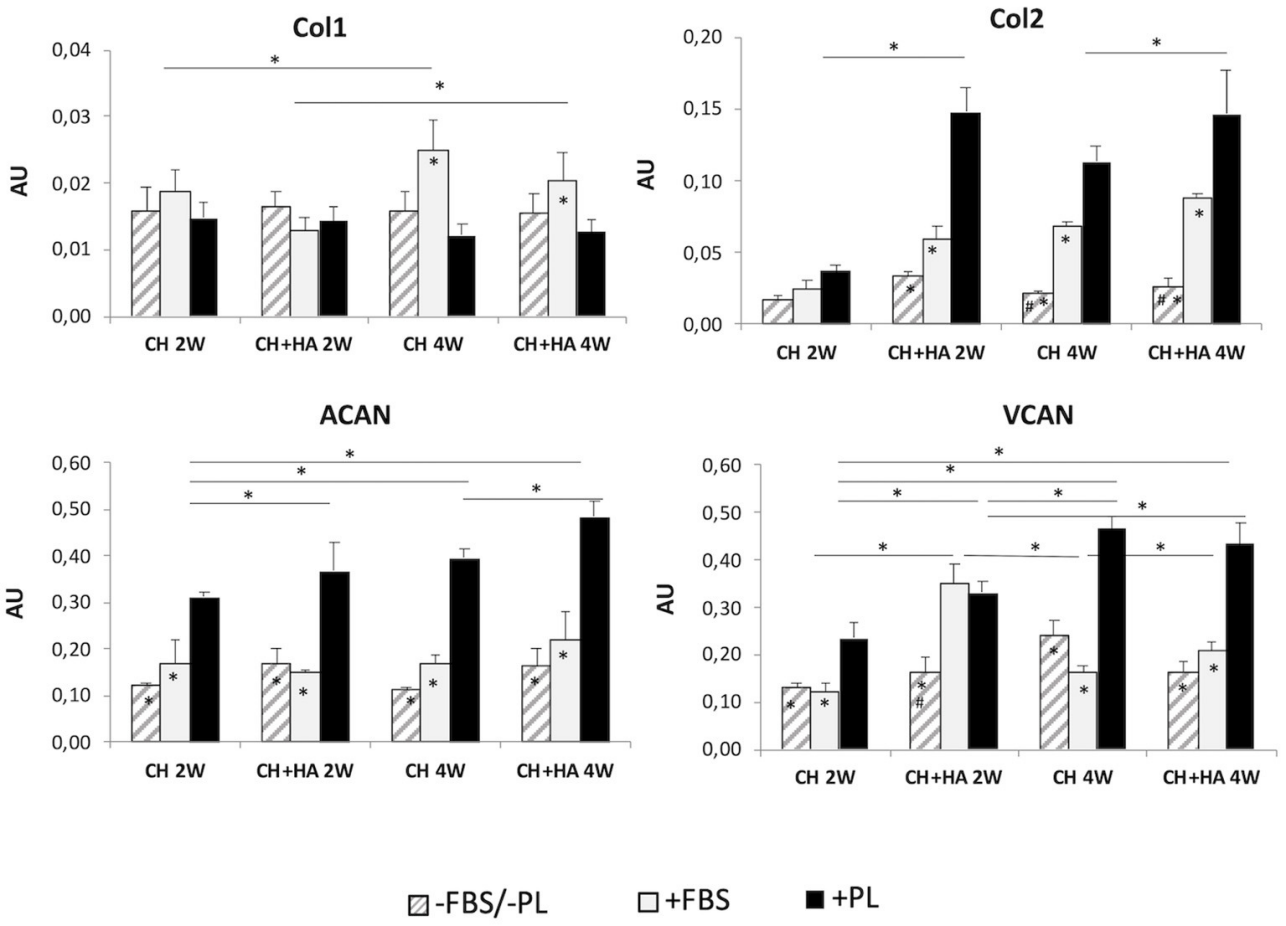
Gene expression of *collagen type I, collagen type II, aggrecan*, and *versican* in chondrocytes cultured in chitosan scaffolds (CH) and chitosan scaffolds prepared with hyaluronic acid (CH+HA) after 2 and 4 weeks in different conditions (–FBS/–PL, +FBS, +PL). Statistical analysis: asterisks above the horizontal lines indicate significant differences of condition during time and among materials (*P < 0.05). Within each material, asterisk indicates significant differences vs. PL and hashtag (#) indicates significant differences vs. FBS (*n* = 9).

The gene expression of *collagen type I* (Figure 6) increased significantly from second to fourth week of culture only in the presence of FBS, particularly for chitosan scaffolds without HA. Moreover, after 4 weeks, the presence of PL reduced the expression of *Col1* with respect to the other conditions. Reduction was significantly only when compared to FBS.

Molecular analysis (Figure 6) revealed a constantly higher expression of *collagen type II* when PL was added, compared with other culture conditions (–FBS/–PL and +FBS), followed by a less pronounced induction of transcription in the presence of FBS. In the FBS/PL-free condition, *collagen type II* expression was generally lower. These differences were not significant in the scaffolds without HA at 2 weeks.

Furthermore, in the presence of PL, the expression of *collagen type II* was increased in scaffolds with HA compared to scaffolds without HA at 2 and 4 weeks of culture. Regarding *aggrecan* (Figure 6), the presence of PL significantly increased *ACAN* gene expression in all the conditions in comparison to that FBS/PL-free and with FBS. Furthermore, when PL was added, the gene expression of *ACAN* increased, in the scaffolds with HA compared to scaffolds without HA at both times, and also increased between the second and fourth weeks in both the types of scaffolds.

After 2 and 4 weeks of culture, the gene expression of *VCAN* (Figure 6) significantly increased when PL was added compared to FBS and –FBS/–PL conditions in all types of scaffold, but only at 2 weeks in the scaffold with HA did FBS produce an increase comparable to PL. The gene expression of *VCAN* at 4 weeks increased only with PL in each type of scaffold, compared to 2 weeks.

#### Gene Expression of Sox9 and Runx2

**Figure 7:**
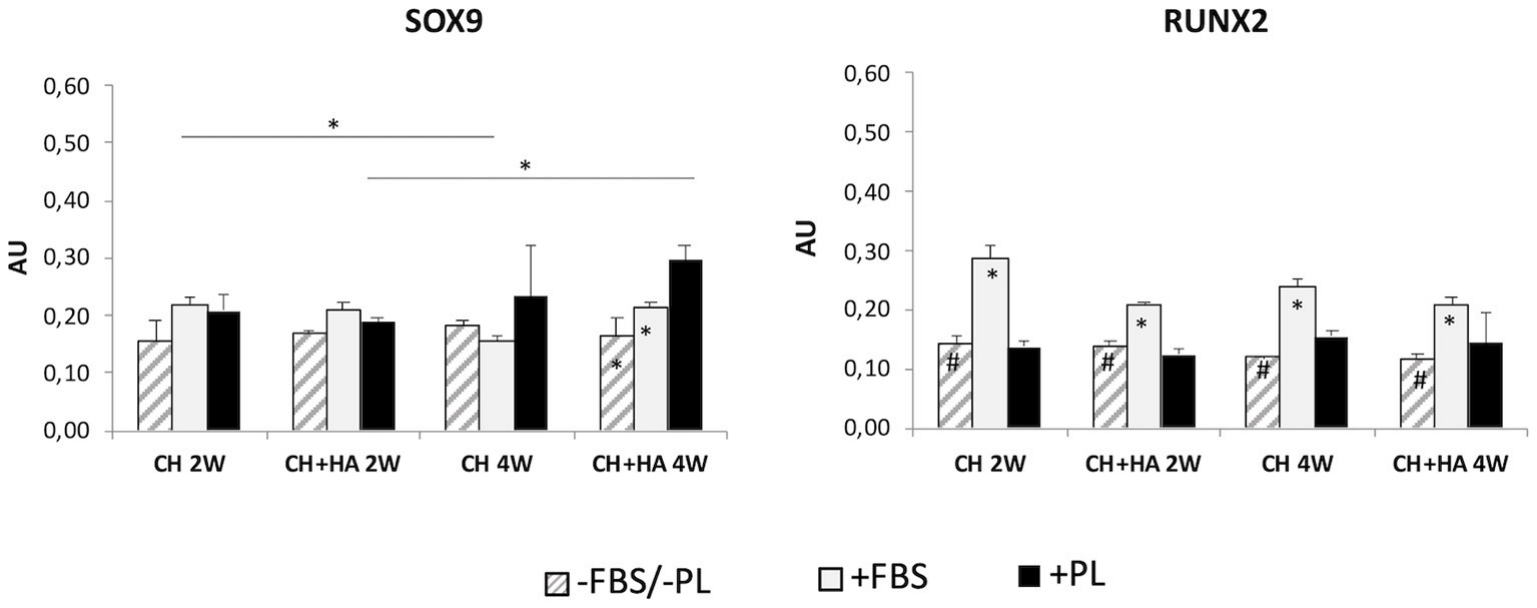
Gene expression of *Sox9* and *Runx2* in chondrocytes cultured in chitosan scaffolds (CH) and chitosan scaffolds prepared with hyaluronic acid (CH+HA) after 2 and 4 weeks in different conditions (–FBS/–PL, +FBS, +PL). Statistical analysis: asterisks above the horizontal lines indicate significant differences of condition during time and among materials (*P < 0.05). Within each material, asterisk indicates significant differences vs. PL and hashtag (#) indicates significant differences vs. FBS (*n* = 9).

A significant increase in *Sox 9* (Figure 7) was observed in scaffolds with HA in the presence of PL after 4 weeks of culture. Furthermore, at 4 weeks, the presence of PL in scaffolds with HA significantly increased *Sox9* gene expression with respect to culture conditions without FBS/PL or with FBS.

Conversely, in scaffolds without HA and with FBS. *Sox9* gene expression was reduced at 4 weeks compared with that after 2 weeks, and *Runx2* (Figure 7) showed increased expression in both scaffolds with and without HA at 2 and 4 weeks only in the presence of FBS, compared with that in the other two culture conditions (without FBS/PL and with PL).

## Discussion

Current opinion on tissue engineering approaches is that better *in vivo* repair can be achieved by combining cells (differentiated cells or MSC) into 3D biomaterial scaffolds made from natural and synthetic polymers with the addition of biological factors or molecules that control or guide cell differentiation (53).

The main role of the scaffold is to support cell colonization, migration, growth, and differentiation for the development and integration of the desired tissue (54). 3D printing has gained popularity in tissue engineering owing to its capability to develop scaffolds with spatial variation in cell distribution or in mechanical properties, and scaffolds built with multiple materials for the cultivation of complex tissues like cartilage (41, 55).

In the present study, the behavior of differentiated chondrocytes (adult horse articular chondrocytes) when cultured in CH-based 3D-printed scaffolds prepared by the method described by Bergonzi (45) was evaluated in terms of their capability to maintain chondrocyte differentiation *in vitro*.

CH hydrogels have demonstrated good capacity to support chondrocyte colonization, vitality, and growth over time in culture (55). This is due to the ability of CH scaffolds to retain a good amount of water and thereby resemble native cartilage, where the water content plays a critical role in the biology and mechanical response of the tissue. An appropriate porosity is a critical characteristic to be considered. A scaffold designed for cartilage tissue engineering must be highly porous to provide a large area for uniform distribution of cells and have a pore size that promotes cell adhesion, cell proliferation, and ECM production (56–58).

In our study, 3D-printed CH scaffolds prepared in saturated NH_3_ gas showed pores having a mean size distribution of 200 μm (45). This porosity demonstrated a good trade-off in terms of cell density and ECM synthesis. It is considered that for successful application, the pore sizes of 100 mm (59) or between 100 and 300 μm can optimize cellular seeding and differentiation while facilitating the inflow and outflow of culture medium and can promote cell nourishment and waste dispersion (60).

HA is a fundamental component of cartilage tissue matrix and its addition to the CH scaffold favored colonization (15, 61). In our study, SEM analysis showed that chondrocytes penetrated inside the 3D structure and colonized the spaces between the fibers. Some cells organized in clusters while others adhered to the surface of scaffold fibers. The chondrocytes cultured with FBS showed evident twofold cell morphology (roundish and fibroblast-like); the roundish morphology was predominant in CH scaffold with HA. In both types of scaffolds, the supplementation with PL showed coupled roundish morphology with a significant amount of filamentous matrix production.

The production of ECM, evaluated using both Masson's trichrome stain and immunostaining for collagen type II, was more abundant in CH scaffolds with HA than in those without HA, particularly when PL was used in alternative to FBS.

Analysis at the molecular level revealed that chondrocytes cultured in 3D conditions maintained phenotypic stability, even though significant differences were observed in the gene expression of ECM components.

*Collagen type I* (which is produced by both pre-chondrocyte mesenchymal cells and osteoblasts) can increase during the dedifferentiation of chondrocyte and OA; this process is also characterized by a disorganization of the ECM and presence of fibrotic repair tissue (62). In our study, Col1 gene expression increased significantly from the second to the fourth week of culture only with supplementation with FBS in CH enriched with HA. It is noteworthy that the presence of PL reduced the expression of Col1 compared with the other conditions.

Moreover, addition of PL increased the gene expression of *Col2, ACAN*, and *VCAN* compared with the other conditions. This was particularly so when the scaffold was prepared with HA.

Together with reduction of *Coll1* and increase of *Coll2* and *ACAN*, the increase of *VCAN* is an important result considering its role in the interaction with the cells and with other ECM molecules to regulate the microenvironment that supports hyaline cartilage formation (63, 64).

*Sox9* is a transcription factor that regulates the differentiation phase of chondrogenesis, while *Runx2* is a transcription factor upregulated in the hypertrophic phenotype and during endochondral ossification (65). With PL treatment and the presence of HA in the scaffold, *Sox9* increased only marginally in the longest experimental time. Regarding *Runx2*, PL maintains low expression levels, which are higher only in the presence of FBS.

In a similar way, molecular investigation verified the morphological phenotype capable of increased colonization and staining of ECM. Moreover, when cultured in 3D scaffolds with HA, the long-term maintenance of chondrocyte phenotype was verified by a reduced gene expression of *Col1* and *Runx2*.

Furthermore, treatment with PL has been confirmed to sustain the proliferation capacity of cultured chondrocytes (29, 66), in a similar way to the FBS.

However, it is also capable of maintaining/promoting chondrocyte differentiation potential in the 3D system (29, 67–69).

In a previous study, we observed that PL succeeded in maintaining a better pattern of gene expression of markers of mature articular chondrocytes compared with FBS, particularly for the percentage ranging from 5 to 10%. In particular, the 5% PL concentration demonstrated to give a better balance between proliferation capacity and maintenance of differentiation (39).

Overall, morphological and biochemical analyses yielded noteworthy results on the effects of PL in combination with the culture in scaffolds enriched with HA.

CH and HA are natural materials and are biocompatible, biodegradable, non-toxic, and inexpensive (14), with chemical characteristics suitable for cartilage tissue engineering. In agreement with previous studies conducted on chondrocytes and stem cells of different origins, the enrichment with HA provide a remarkable microenvironment for the culture system (61, 70–72).

Furthermore, considering that FBS is not recommended for *in vivo* use (29), PL can be suggested as a good alternative.

In conclusion, the use of 3D CH scaffold when PL is added as a substitute for FBS promotes the gene expression of most relevant ECM components. This indicates that this cultivation system enables a better maintenance of chondrocyte phenotype, highlighting an effective trade-off between proliferation and differentiation (73) and should be considered as a useful option in future application. Results of the present study are encouraging, and the validation of the construct with preclinical studies in both ectopic and mechanically loaded applications should continue.

## Data Availability Statement

The raw data supporting the conclusions of this article will be made available by the authors, without undue reservation.

## Author Contributions

PB, ED, FR, and RB contributed to conception and design of the study. ED, LE, AB, CB, MP, RR, MA, VCa, VCo, and PB performed laboratory preparation and testing organized the database. RS and PM performed the statistical analysis. PB and ED wrote the first draft of the manuscript. FR, RB, PB, and RS wrote sections of the manuscript. All authors contributed to manuscript revision, read, and approved the submitted version.

## Conflict of Interest

The authors declare that the research was conducted in the absence of any commercial or financial relationships that could be construed as a potential conflict of interest.
